# VAAFT plus FiLaC™: a combined procedure for complex anal fistula

**DOI:** 10.1007/s10151-021-02411-0

**Published:** 2021-01-21

**Authors:** Y.-B. Yao, C.-F. Xiao, Q.-T. Wang, H. Zhou, Q.-J. Dong, Y.-Q. Cao, C. Wang

**Affiliations:** Department of Anorectal Surgery, Longhua Hospital, Shanghai University of Traditional Chinese Medicine, Shanghai, 200030 China

**Fig. 1:**
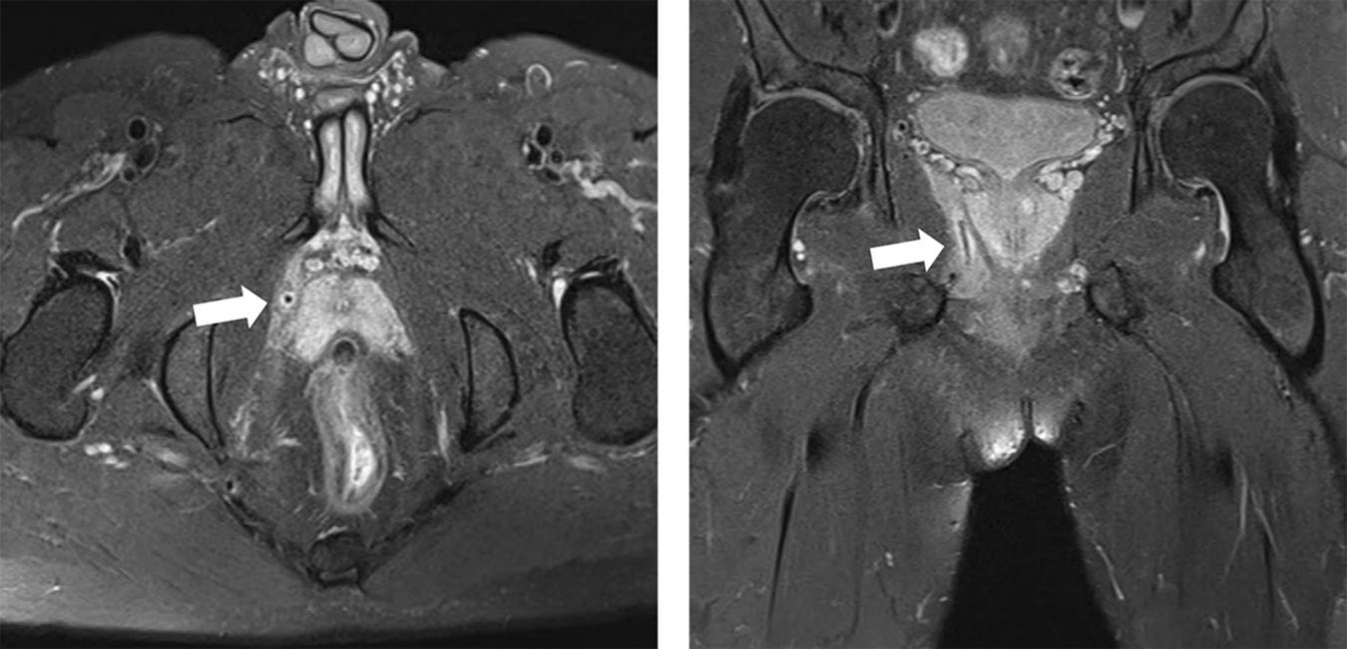
Preoperative perianal magnetic resonance imaging shows the long fistula tract (white arrow) located near the prostate and under the levator ani muscle

**Fig. 2:**
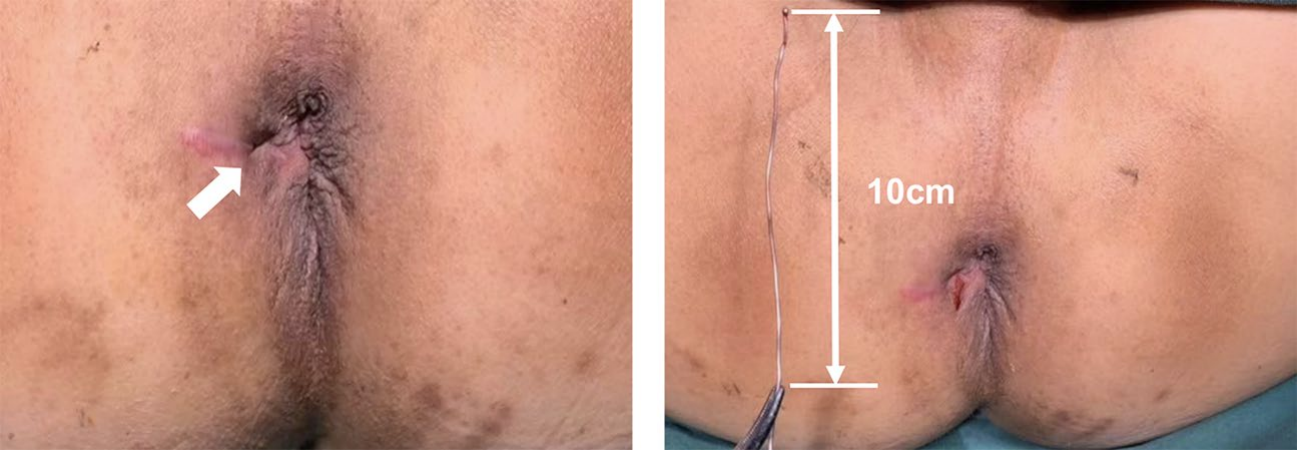
Identification of the fistula tract during the operation. The patient was placed in a lithotomy position under subarachnoid anesthesia. There was a scar and an external opening at 2 cm from the anal verge (white arrow). Exploration with the probe revealed that the fistula tract was about 10 cm long

**Fig. 3:**
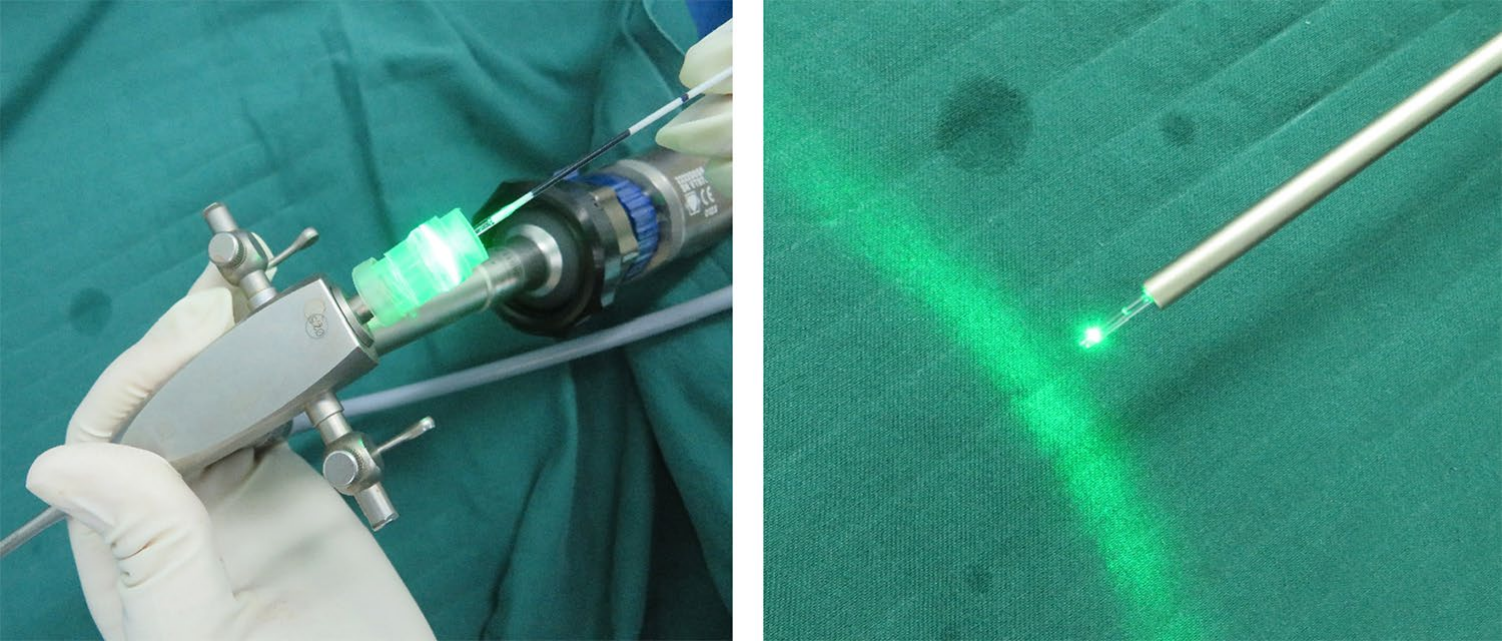
Placing laser fibre into the fistuloscope. We replaced unipolar the electrode of VAAFT (Karl Storz GmbH, Tuttlingen, Germany), with the radial laser probe of FiLaC™ (Biolitec Biomedical Technology GmbH, Jena, Germany)

**Fig. 4:**
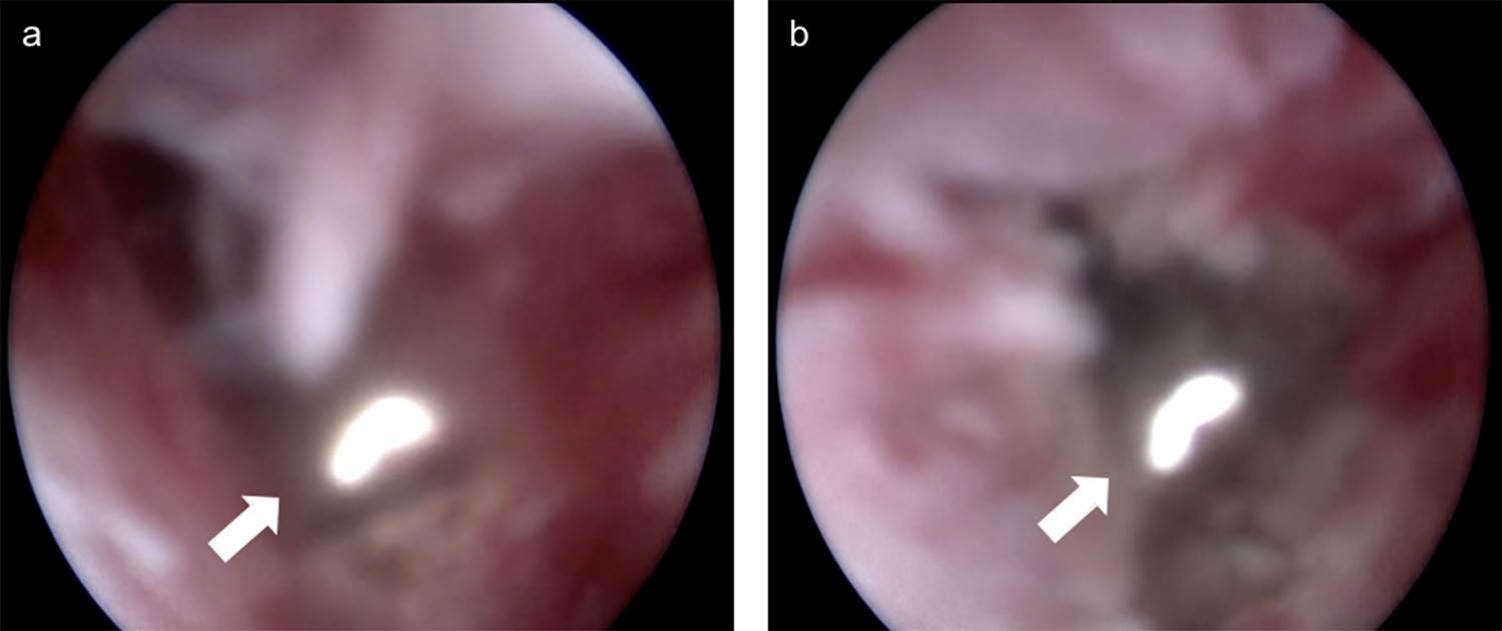
Direct vision was provided by the fistuloscope while the radial laser probe (14 W power at wavelength of 1470 nm) was shrinking and sealing the tract (White arrow: radial laser probe). **a** BEFORE laser closure. **b** The fistula tract had obviously shrunk after laser closure

**Fig. 5:**
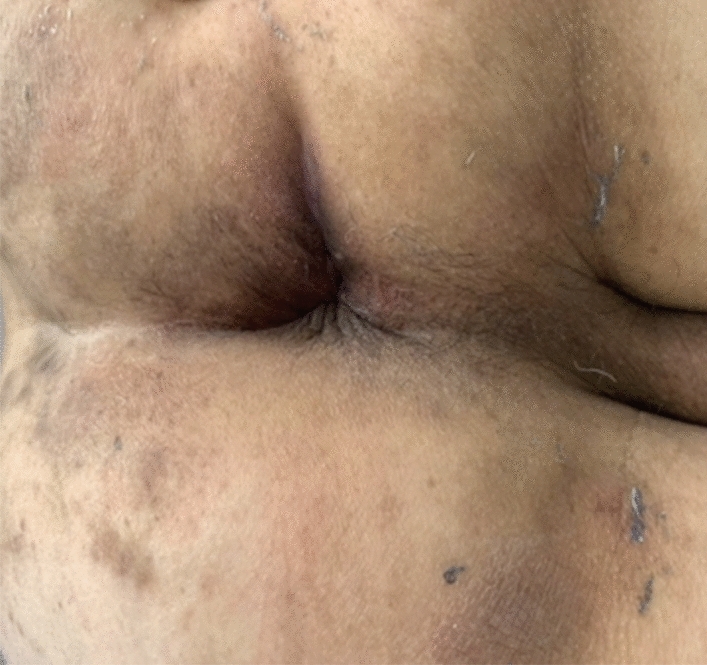
Wound healing 2 months after the operation

**Fig. 6:**
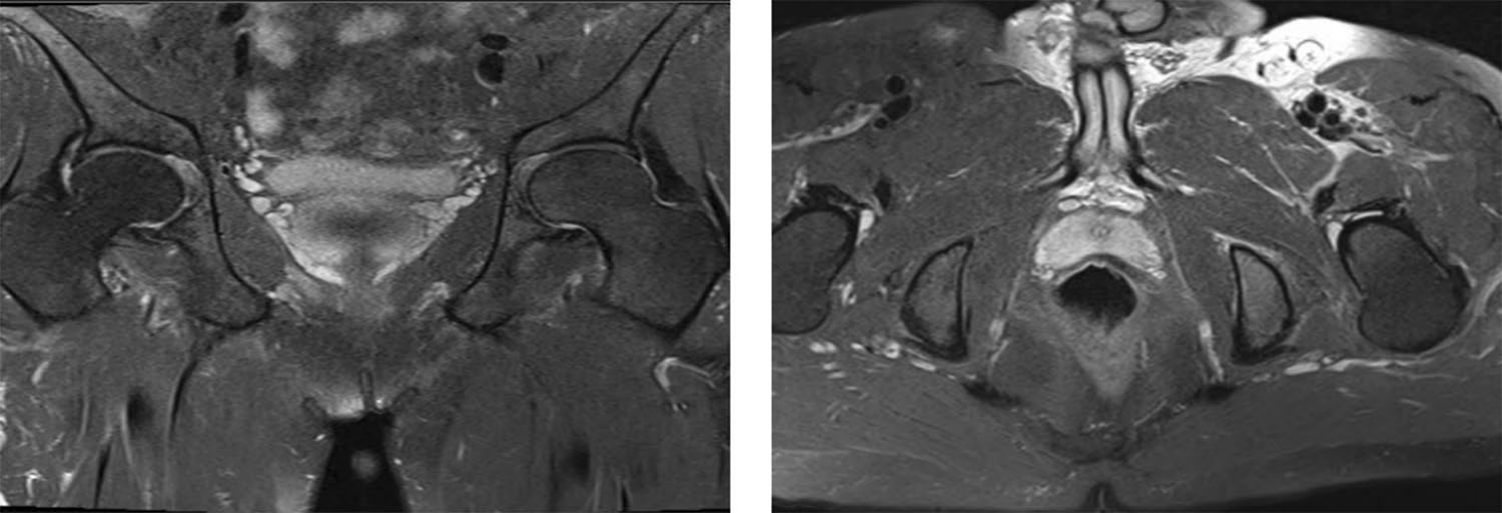
Perianal magnetic resonance imaging (MRI) 4 months after the operation. The long fistula tract was disappeared. The perianal MRI shows excellent healing

The treatment of complex anal fistula is a challenge, because inappropriate surgery may cause fecal incontinence. Video-assisted anal fistula treatment (VAAFT) and fistula tract laser closure (FiLaC™) are both minimally invasive and sphincter-saving techniques for treating anal fistula. VAAFT can treat fistula tracts under direct vision and FiLaC™ can achieve circular closure of fistula tracts. VAAFT plus FiLaC™ combines the advantages of two technologies and is a promising procedure for complex anal fistula (Figs. 1, 2, 3, 4, 5, 6).

